# Diacylglycerol acyltransferase 1/2 inhibition induces dysregulation of fatty acid metabolism and leads to intestinal barrier failure and diarrhea in mice

**DOI:** 10.14814/phy2.14542

**Published:** 2020-08-12

**Authors:** Kosuke Takemoto, Yumiko Fukasaka, Ryo Yoshimoto, Hirohide Nambu, Hideo Yukioka

**Affiliations:** ^1^ Drug Discovery & Disease Research Laboratory Shionogi & Co., Ltd Osaka Japan; ^2^ Laboratory of Veterinary Pathology, Joint Faculty of Veterinary Medicine Yamaguchi University Yamaguchi Japan

**Keywords:** barrier dysfunction, diacylglycerol acyltransferase, diarrhea, fatty acids, triglycerides

## Abstract

The intestinal metabolism and transport of triacylglycerol (TAG) play a critical role in dietary TAG absorption, and defects in the process are associated with congenital diarrhea. The final reaction in TAG synthesis is catalyzed by diacylglycerol acyltransferase (DGAT1 and DGAT2), which uses activated fatty acids (FA) as substrates. Loss‐of‐function mutations in DGAT1 cause watery diarrhea in humans, but mechanisms underlying the relationship between altered DGAT activity and diarrhea remain largely unclear. Here, the effects of DGAT1 and DGAT2 inhibition, alone or in combination, on dietary TAG absorption and diarrhea in mice were investigated by using a selective DGAT1 inhibitor (PF‐04620110) and DGAT2 inhibitor (PF‐06424439). Simultaneous administration of a single dosing of these inhibitors drastically decreased intestinal TAG secretion into the blood circulatory system and TAG accumulation in the duodenum at 60 min after lipid gavage. Under 60% high‐fat diet (HFD) feeding, their repeated simultaneous administration for 2 days induced severe watery diarrhea and occasionally led to death. The diarrhea was accompanied by enhanced fecal FA excretion, intestinal injury and barrier failure. DGAT1 or DGAT2 inhibition alone did not induce the phenotypic changes observed in DGAT1/2 inhibitor‐treated mice. The results demonstrate that DGAT1/2 inhibition alters TAG absorption and results in watery diarrhea in mice. DGAT1/2 inhibition‐induced diarrhea may be caused by intestinal barrier dysfunction due to dysregulation of the cytotoxic FA metabolism. These findings suggest that DGAT‐mediated intestinal TAG synthesis is a vital step for maintaining intestinal barrier integrity under HFD feeding.

## Graphical abstract

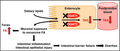

**Abbreviations**
CM: chylomicrons
DAG: diacylglycerol
DGAT: diacylglycerol acyltransferase
ELISA: enzyme‐linked immunosorbent assay
FA: fatty acids
FD4: fluorescein isothiocyanate‐dextran
HFD: high fat diet
MAG: monoacylglycerol
PGE_2_: prostaglandin E_2_
SAR1B: secretion‐associated Ras‐related GTPase 1B
*SEM*: standard error of the mean
TAG: triacylglycerol
TLC: thin‐layer chromatography

## 1 INTRODUCTION

The intestinal metabolism and transport of triacylglycerol (TAG) play a key role in the regulation of dietary TAG absorption (D'Aquila, Hung, Carreiro, & Buhman, 1861; Yen, Nelson, & Yen, 2015). Dietary lipids consist mostly of TAG, which is hydrolyzed to 2‐monoacylglycerol (2‐MAG) and fatty acids (FA) in the intestinal lumen Yen et al., 2015; Mattson & Volpenhein, 1964). These hydrolysis products are taken up by enterocytes, absorptive cells of the small intestine, and resynthesized to TAG (Yen et al., (2015); Mattson & Volpenhein, 1964). The resynthesized TAG is either incorporated into chylomicrons (CM) to be secreted into the blood through the lymphatic system or accumulated temporarily in enterocytes (Yen et al., 2015).

Functional defects in the cellular processes which regulate TAG absorption are associated with congenital diarrheal disorders (Berriot‐Varoqueaux, Aggerbeck, Samson‐Bouma, & Wetterau, 2000; Haas et al., 2012; Jones et al., 2003; Overeem, Posovszky, Rings, & Giepmans, 2016). Mutations in the microsomal triglyceride transfer protein gene and the secretion‐associated Ras‐related GTPase 1B (SAR1B) gene, both of which regulate transport of TAG in enterocytes, lead to lipid malabsorption and steatorrhea (Berriot‐Varoqueaux et al., 2000; Jones et al., 2003; Overeem et al., 2016). The phenotype is considered to be caused by excessive TAG accumulation in enterocytes (D'Aquila et al., 1861; Georges et al., 2011; Iqbal, Parks, & Hussain, 2013). Interestingly, rare loss‐of‐function‐mutations in the diacylglycerol acyltransferase 1 (DGAT1) gene, which catalyzes TAG synthesis using activated FA and diacylglycerol (DAG) in enterocytes, lead to watery diarrhea in humans but not to steatorrhea (Haas et al., 2012). However, the underlying mechanisms of DGAT1 mutation leading to diarrhea in human are unknown since mice deficient in DGAT1 gene are healthy (Buhman et al., 2002; Smith et al., 2000).

In this study, we investigated the effects of DGAT1 and DGAT2 inhibition, alone or in combination, on dietary TAG absorption and diarrhea in mice since two DGAT isoforms, DGAT1 and DGAT2, are highly expressed in the mouse small intestine (Cases et al., 1998, 2001; Haas et al., 2012). We found that simultaneous administration of selective DGAT1 and DGAT2 inhibitors (Dow et al., 2011; Futatsugi et al., 2015) alters TAG absorption processes and, under high‐fat diet (HFD) feeding, leads to watery diarrhea whereas either inhibition alone did not lead to diarrhea. Moreover DGAT1/2 inhibition‐induced diarrhea was accompanied by increases in fecal FA and intestinal inflammatory markers, such as intestinal prostaglandin E_2_ (PGE_2_) and fecal calprotectin (Summerton, Longlands, Wiener, & Shreeve, 2002; Wiercińska‐Drapało, Flisiak, & Prokopowicz, 2001), and intestinal barrier failure. These findings imply that altered FA metabolism and inflammatory responses may be associated with the diarrhea.

## 2 MATERIALS AND METHODS

### 2.1 Animals and drugs

All animal studies were approved by the Institutional Animal Care and Use Committee at Shionogi & Co., Ltd. All experimental procedures were conducted in a facility accredited by the Association for Assessment and Accreditation of Laboratory Animal Care International. Six‐week‐old male C57BL/6 mice were obtained from CLEA Japan (Tokyo, Japan) and housed in groups of four or five per cage under controlled environmental conditions (24 ± 2°C; 50 ± 20% relative humidity; 12‐hr light/dark cycle, lights on at 8:00 a.m.). The group‐housed mice were maintained on 60% HFD (58Y1; TestDiet, St. Louis, MO, USA) for 4 weeks and caged individually before being used in experiments. The fat composition of the diet consisted of 47 g/kg linoleic acid, 3.9 g/kg linolenic acid, 0.6 g/kg arachidonic acid, 3.9 g/kg omega‐3 fatty acids, 140 g/kg monounsaturated fatty acids, and 136.8 g/kg saturated fatty acids. Throughout this study, the mice had free access to food and tap water unless otherwise stated.

A DGAT1 inhibitor (PF‐04620110) was purchased from MedChem Express (Monmouth Junction, NJ, USA), and a DGAT2 inhibitor (PF‐06424439) was purchased from Sigma‐Aldrich (St. Louis, MO, USA). The DGAT1 inhibitor (3 mg/kg) or DGAT2 inhibitor (7.5 mg/kg) was suspended in 0.5% w/v hydroxypropylmethylcellulose aqueous solution (Shin‐Etsu Chemical, Tokyo, Japan) and orally administered to mice at a volume of 10 ml/kg body weight. The dose of the DGAT1 inhibitor was selected to produce maximal inhibitory effects on intestinal TAG secretion in mice (Figure S1a). The dose of the DGAT2 inhibitor was selected to produce maximal additional inhibitory effects on intestinal TAG secretion in mice in the presence of the DGAT1 inhibitor because the DGAT2 inhibitor alone did not show inhibitory effects (Figure S1b, c).

### 2.2 Analysis of dietary TAG absorption

After 4 weeks of 60% HFD feeding, the mice were made to fast for 24 hr prior to administration of vehicle or the DGAT inhibitors. At 15 min after the administration, 500 mg/kg of Pluronic F‐127 (Sigma‐Aldrich) was intraperitoneally injected to inhibit clearance of plasma TAG. At 15 min after the injection, 50 µ Ci/kg of [carboxyl‐carbon‐14] triolein (Perkin Elmer, Waltham, MA, USA) in 20% lipid emulsion (Intralipos Injection 20%; Otsuka Pharmaceutical Factory, Tokushima, Japan) was given by oral gavage. Blood and duodenal samples were collected under anesthesia 60 min after the oral gavage. Plasma samples were obtained from the blood samples by centrifugation at 5,000 × g for 10 min at 20°C, and plasma radioactivity was quantified by measuring ionizing radiation with a MicroBeta TRILUX counter (PerkinElmer). The duodenal samples were weighed and homogenized in 1 ml of ice‐cold H_2_O. The homogenates were mixed vigorously with 5 ml of chloroform/methanol (3:2, v/v) and 1 ml of 1 M NaCl, and the mixtures centrifuged at 840 × g for 20 min at room temperature separated into two phases. After removing the upper phase, the lower chloroform phase containing lipids was evaporated under a nitrogen stream. The dried residue was dissolved in 1 ml of hexane, and the dissolved lipids were separated by thin‐layer chromatography (TLC). The radioactivity of TAG, FA, and DAG fractions on a TLC plate was quantified with a FLA‐3000 imaging system (Fuji Film, Tokyo, Japan).

### 2.3 Sample preparation for analysis of diarrhea, fecal lipid excretion, histology, and inflammatory markers

After 4 weeks of 60% HFD feeding, the vehicle or the DGAT inhibitors were orally administered twice daily to mice for 2 or 3 days. All feces were collected during the period and processed for analysis of fecal lipid excretion and diarrhea or stored at −80°C until biochemical analysis. Small intestinal tissues were dissected under anesthesia 4 hr after the final dose of the DGAT inhibitors and cut into three segments corresponding to the duodenum, jejunum and ileum. After washing with ice‐cold saline, the small intestinal samples were fixed in 10% formalin for 24 hr until histological analysis or stored at −80°C until biochemical analysis.

### 2.4 Analysis of diarrhea scores and fecal water content

The severity of the diarrhea was assessed by scoring each fecal sample according to the following scale: 0 (no diarrhea): normal feces; 1 (very mild diarrhea): moist feces, and no sign of soiling around the anus; 2 (mild diarrhea): moist and soft feces, and some signs of soiling around the anus; 3 (diarrhea): unformed feces, and considerable soiling around the anus; 4 (severe watery diarrhea): liquid feces, and considerable soiling around the anus as previously described by Pearson et al. (Pearson et al., 2013).

The fecal samples were lyophilized after measurement of wet weight, and then the dry weight was measured. Water content was calculated by subtracting the dry fecal weight from the wet fecal weight.

### 2.5 Analysis of fecal lipid excretion

The fecal lipids were extracted from the fecal samples by the above described methods of lipid extraction. The dried residue was dissolved in 1 ml of isopropanol, and the dissolved lipids were used to determine the levels of fecal FA and TAG by enzymatic methods (Sekisui Medical, Tokyo, Japan) with a Hitachi 7,170 autoanalyzer (Hitachi, Tokyo, Japan).

### 2.6 Histological analysis

The small intestinal samples fixed with formalin were routinely processed and embedded in paraffin wax. The intestinal sections of 3 µ m thickness were prepared and stained with hematoxylin and eosin.

### 2.7 Analysis of intestinal inflammatory markers

The small intestinal samples were weighed and homogenized in 10 volumes of ice‐cold lysis buffer containing 100 mM phosphate buffer (pH 7.4), 1 mM EDTA and 10 µ M indomethacin. The homogenates were centrifuged at 8,000 × g for 10 min at 4°C to remove tissue debris, and the supernatants were used to determine intestinal PGE_2_ levels with a PGE_2_ enzyme‐linked immunosorbent assay (ELISA) kit (Cayman Chemical, Ann Arbor, MI, USA). The protein content of each sample was quantified with a BCA^TM^ Protein Assay Kit (Thermo Fisher Scientific, Rockford, IL, USA).

The fecal samples were weighed, and fecal calprotectin levels were determined with a S100A8/S100A9 ELISA kit (Immunodiagnostik, Bensheim, Germany) according to the manufacturer's instructions.

### 2.8 Analysis of intestinal epithelial barrier function

After 4 weeks of 60% HFD feeding, the vehicle or the DGAT inhibitors were orally administered twice daily to mice for 2 days. Fluorescein isothiocyanate‐dextran (FD4; Sigma Aldrich) was diluted to 22 mg/ml in phosphate buffered saline and orally administered to the mice in a volume of 10 ml/kg body weight immediately after the final dose of the DGAT inhibitors. Blood samples were collected 5 hr after the administration of FD4, and plasma samples were obtained from the blood samples by centrifugation at 6,000 × g for 10 min at 4°C. Plasma FD4 concentration was quantified by measuring fluorescence intensity using a VERSAmax microplate reader (Molecular Devices, Sunnyvale, CA, USA) with excitation at 490 nm and emission at 535 nm.

### 2.9 Statistical analysis

All results are presented as the mean ± standard error of the mean (*SEM*). Data were analyzed by two‐tailed Welch's *t* test or Dunnett's multiple comparison test. Statistical analyses were performed by the SAS Version 9.4 for Windows (SAS Institute, Cary, NC, USA). A value of *p* < .05 was considered statistically significant.

## 3 RESULTS

### 3.1 Roles of two DGAT isoforms in dietary TAG absorption

**Figure 1:**
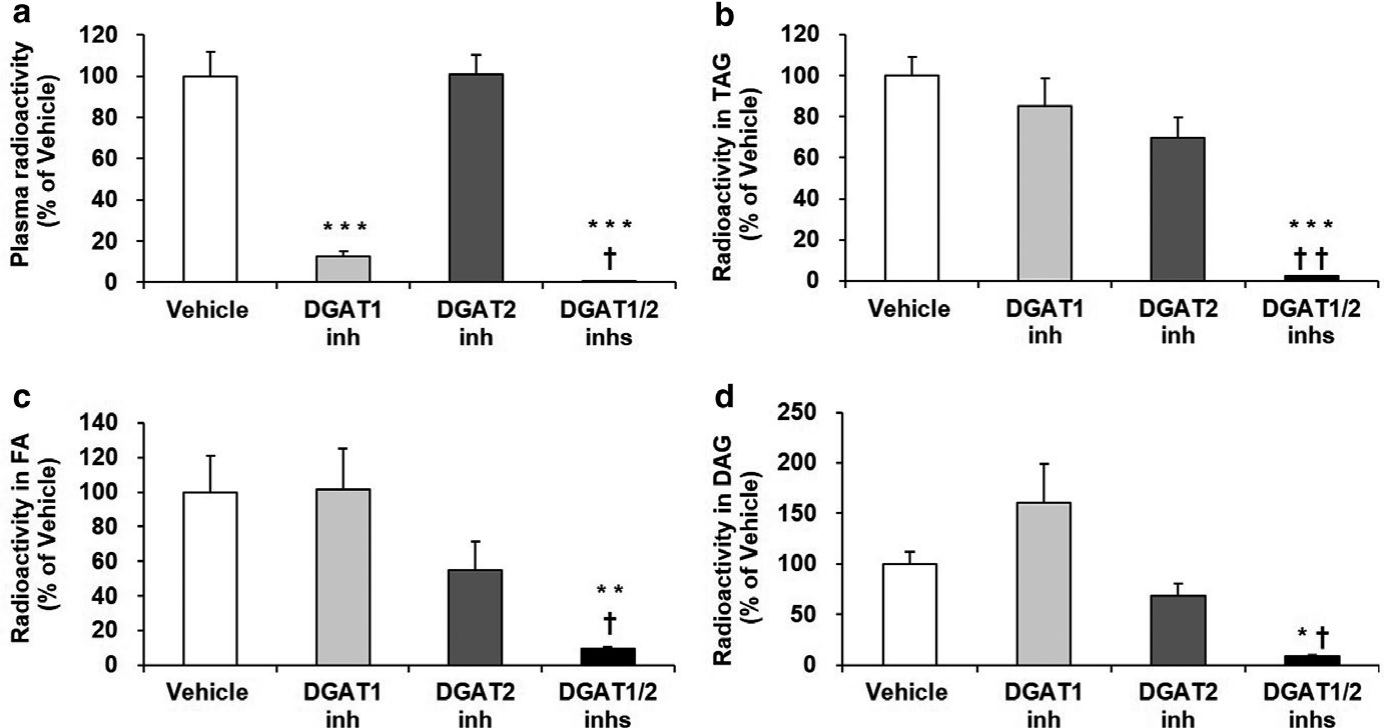
Effects of DGAT inhibitors on radioactivity of plasma (A) and duodenal lipid fractions (B‐D) after oral gavage of [^14^C] TAG to mice. A DGAT1 inhibitor (3 mg/kg) and DGAT2 inhibitor (7.5 mg/kg), alone or in combination, were orally administered to fasted mice prior to oral gavage of lipid emulsion containing 50 µCi/kg of [^14^C] TAG. Radioactivity in plasma and duodenal samples was measured at 60 min after the oral gavage. The results are expressed as the mean ± *SEM* (*n* = 4). *p < .05, **p < .01, ***p < .001 versus Vehicle group (Dunnett's multiple‐comparison test). ^†^ *p* < .05, ^††^ *p* < .01 versus DGAT1 inhibitor‐treated group (Welch's *t* test)

To investigate the roles of DGAT1 and DGAT2 in TAG absorption, we examined the effects of the DGAT1 inhibitor (1 mg/kg) and the DGAT2 inhibitor (7.5 mg/kg) on plasma radioactivity at 60 min after oral gavage of [^14^C] TAG to mice. The DGAT1 inhibitor given alone significantly decreased plasma radioactivity compared with vehicle, while the DGAT2 inhibitor given alone did not affect plasma radioactivity (Figure 1a). Simultaneous administration of the DGAT1 and DGAT2 inhibitors significantly decreased plasma radioactivity compared with the DGAT1 inhibitor given alone. More than 96% of the measured plasma radioactivity was identified as [^14^C] TAG by TLC analysis (data not shown).

We next examined the effects of the DGAT inhibitors on levels of duodenal radiolabeled TAG and DGAT substrates at 60 min after oral gavage of [^14^C] TAG to mice. While the DGAT1 or DGAT2 inhibitor given alone did not affect levels of duodenal radiolabeled TAG, FA, and DAG compared with the vehicle, their simultaneous administration significantly decreased their levels (Figure 1b‐d).

### 3.2 Watery diarrhea and enhanced FA excretion in DGAT1/2 inhibitor‐treated mice

**Figure 2:**
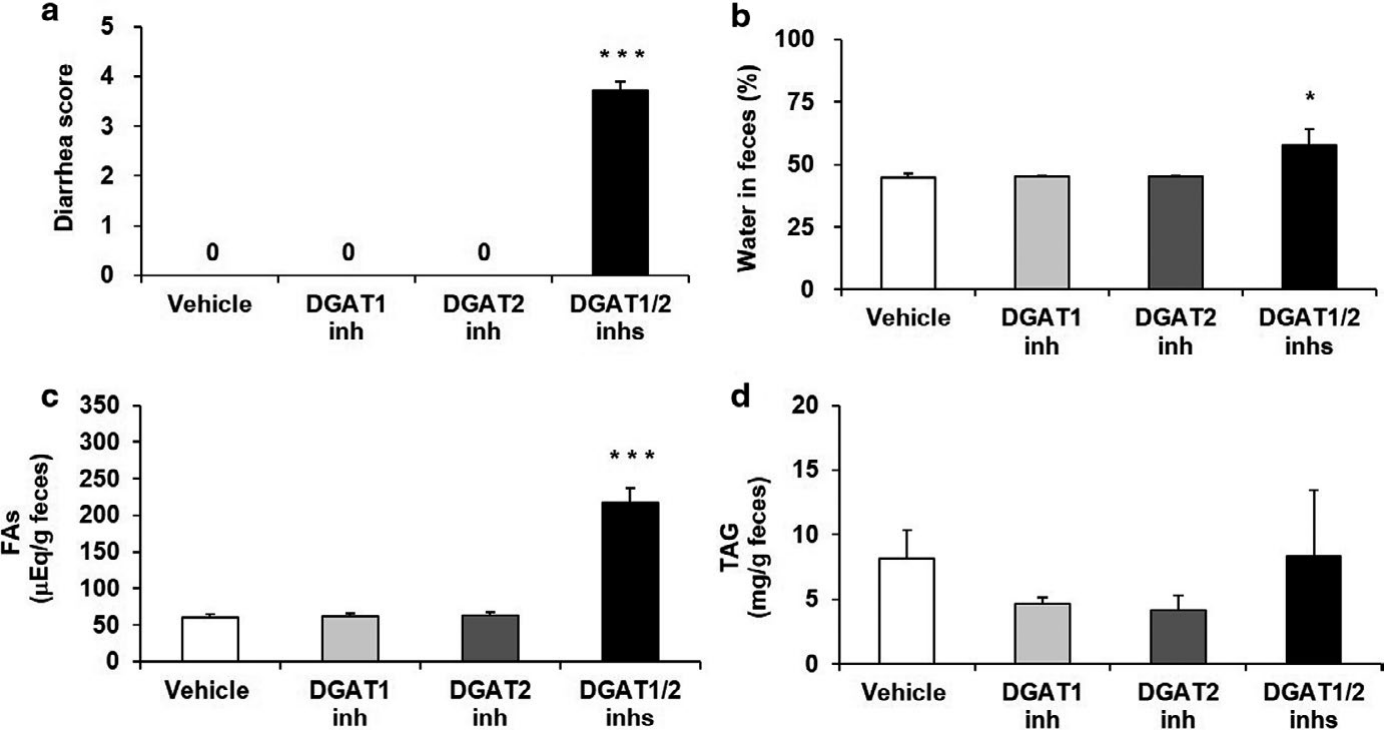
Effects of DGAT inhibitors on diarrhea scores (A), fecal water content (B) and fecal levels of FA (C) and TAG (D) in 60% HFD‐fed mice. A DGAT1 inhibitor (3 mg/kg) and DGAT2 inhibitor (7.5 mg/kg), alone or in combination, were orally administered twice daily to 60% HFD‐fed mice for 2 days. All feces were collected during the period, and diarrhea scores were evaluated by measuring the softness and appearance of each fecal sample. Water content was calculated by subtracting the dry fecal weight from the wet fecal weight. Fecal levels of FA and TAG were determined by enzymatic methods. The results are expressed as the mean ± *SEM* (*n* = 7). *p < .05, ***p < .001 versus Vehicle group (Dunnett's multiple‐comparison test)

Under HFD feeding, DGAT1 or DGAT2 inhibitor‐treated mice appeared healthy. After repeated simultaneous administration of the DGAT inhibitors, all mice exhibited diarrhea (Figure 2a), and one out of the five mice died (data not shown). Simultaneous administration of the DGAT inhibitors, but neither of them alone, significantly increased fecal water content compared with the vehicle (Figure 2b). Severity of diarrhea depended on lipid content of the diets (Figure S2).

To determine how DGAT1/2 inhibition affects lipid distribution, we examined the effects of the DGAT inhibitors on fecal lipid excretion in HFD‐fed mice. Simultaneous administration of the DGAT inhibitors significantly increased levels of fecal FA but not of fecal TAG compared with the vehicle (Figure 2c,d), whereas DGAT1 or DGAT2 inhibitor given alone had no effect.

To further understand relationship between diarrhea and changes in DGAT activity, we examined dose‐dependent effects of DGAT inhibitors in mice. Simultaneous administration of a lower‐dose DGAT1 (0.03 mg/kg) inhibitor, which did not produce maximal effects on intestinal TAG secretion, and the DGAT2 inhibitors significantly decreased plasma radioactivity but to a lesser extent as the DGAT1 inhibitor (1 mg/kg) given alone (Figure S3). The low‐dose simultaneous administration significantly increased diarrhea scores and tends to increase the fecal FA levels under HFD feeding (Figure S4a, S4b).

### 3.3 Pathological changes and altered inflammatory responses in DGAT1/2 inhibitor‐treated mice

**Figure 3:**
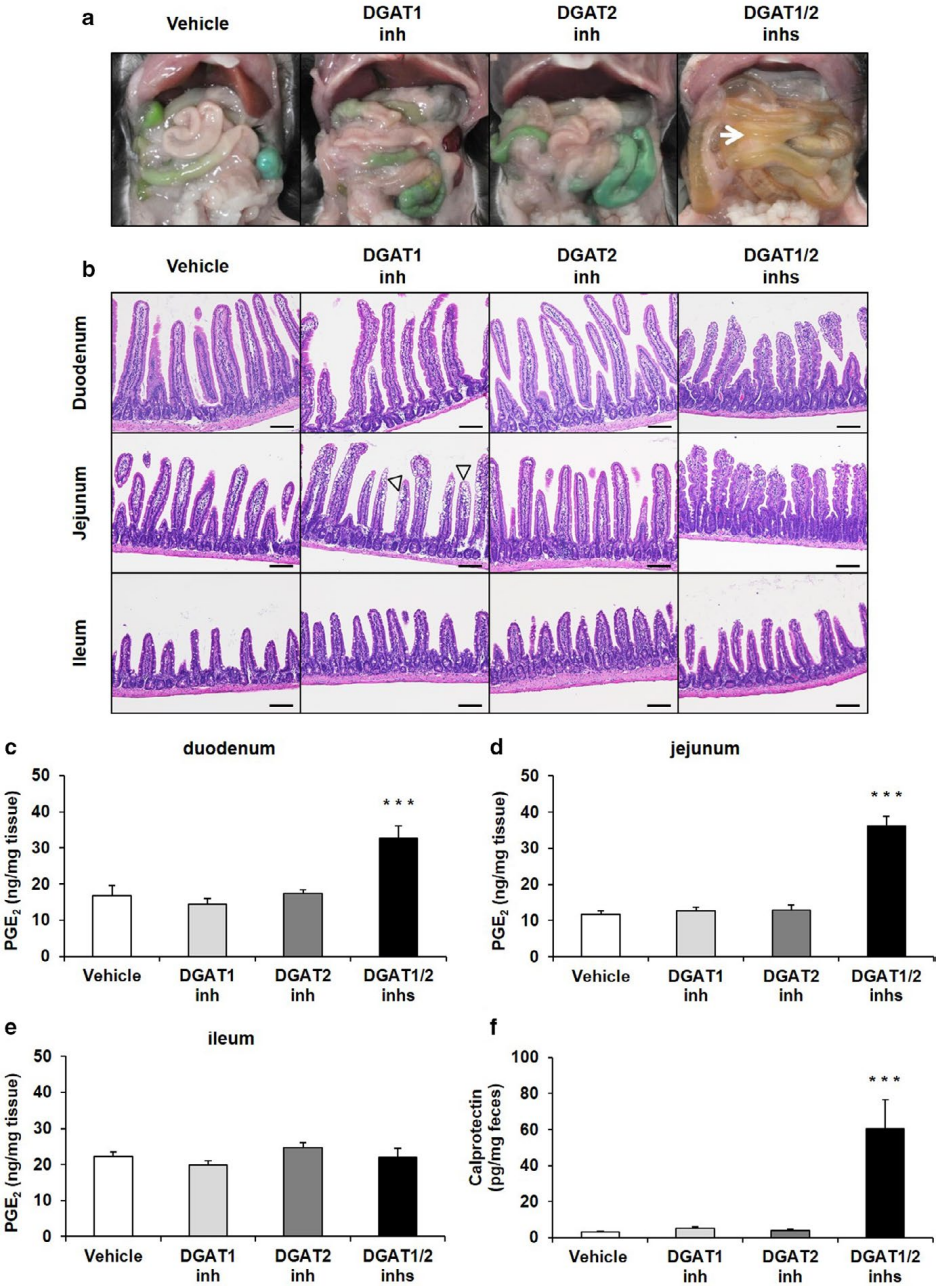
Effects of DGAT inhibitors on gross appearance (A), histology of the small intestine (B), intestinal PGE_2_ levels (C‐E) and fecal calprotectin levels (F) in 60% HFD‐fed mice. A DGAT1 inhibitor (DGAT1 inh; 3 mg/kg) and DGAT2 inhibitor (DGAT2 inh; 7.5 mg/kg), alone or in combination (DGAT1/2 inhs), were orally administered twice daily to 60% HFD‐fed mice for 3 days, and all feces were collected during the period. The mice were euthanized 4 hr after the final dose of the DGAT inhibitors, and then small intestinal samples were dissected. An arrow indicates a fluid‐filled small intestine. Sections of the small intestine were stained with hematoxylin and eosin. Arrowheads indicate a large number of intracytoplasmic vacuoles. Scale bars represent 100 µm. Levels of intestinal PGE_2_ and fecal calprotectin were determined with ELISA kits. The results are expressed as the mean ± *SEM* ((C‐E) *n* = 7; (F) *n* = 4 mice for DGAT1/2 inhs group and *n* = 7 mice for all other groups). ***p < .001 versus Vehicle group (Dunnett's multiple‐comparison test)

To explore the DGAT1/2 inhibition‐induced mechanisms leading to the watery diarrhea, we examined the effects of the DGAT inhibitors on gross appearance and histology of the small intestinal tissue in HFD‐fed mice. Simultaneous administration of the DGAT inhibitors induced gastrointestinal disturbances such as a fluid‐filled small intestine (Figure 3a). Furthermore, marked crypt hyperplasia and shortening of the villi were observed in the duodenum and jejunum, but not in the ileum, of the DGAT1/2 inhibitor‐treated mice (Figure 3b). The intestinal pathological changes were not observed in the DGAT1 or DGAT2 inhibitor‐treated mice. A large number of intracytoplasmic vacuoles were observed in the jejunum of the DGAT1 inhibitor‐treated mice but not in the DGAT2 inhibitor‐treated mice or DGAT1/2 inhibitor‐treated mice (Figure 3b).

To investigate the involvement of inflammation in the intestinal pathological changes induced by DGAT1/2 inhibition, we examined the effects of the DGAT inhibitors on levels of intestinal PGE_2_ and fecal calprotectin in HFD‐fed mice. Simultaneous administration of the DGAT inhibitors significantly increased PGE_2_ levels in the duodenum and jejunum, but not in the ileum, and calprotectin levels in the feces compared with the vehicle (Figure 3c‐f). No increases in inflammatory markers were observed in DGAT1 or DGAT2 inhibitor‐treated mice. The simultaneous administration of the lower‐dose DGAT1 (0.03 mg/kg) and DGAT2 inhibitors tend to increase PGE_2_ levels in the jejunum and calprotectin levels in the feces (Figure S4c,d).

### 3.4 Intestinal barrier failure in DGAT1/2 inhibitor‐treated mice

**Figure 4:**
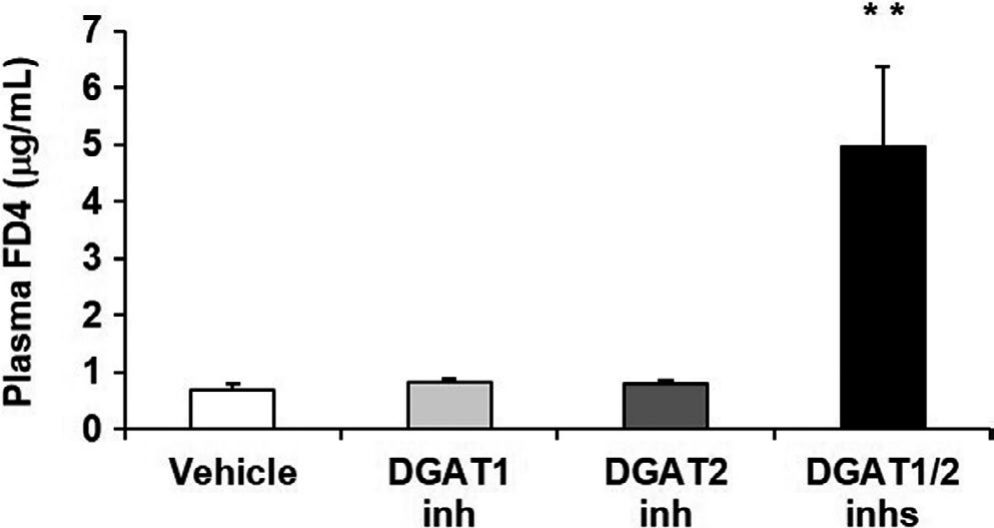
Effects of DGAT inhibitors on intestinal permeability of FD4 in 60% HFD‐fed mice. A DGAT1 inhibitor (3 mg/kg) and DGAT2 inhibitor (7.5 mg/kg), alone or in combination, were orally administered twice daily to 60% HFD‐fed mice for 2 days. FD4 (22 mg/kg) was orally administered to the mice immediately after the final dose of the DGAT inhibitors. Blood samples were collected 5 hr after the administration of FD4, and plasma FD4 concentration was quantified by measuring fluorescence intensity. The results are expressed as the mean ± *SEM* (*n* = 4 mice for DGAT1/2 inhs group and *n* = 5 mice for all other groups). **p < .01 versus Vehicle group (Dunnett's multiple‐comparison test)

To investigate the cause of the fluid‐filled small intestine observed in the DGAT1/2 inhibitor‐treated mice, we examined the intestinal epithelial barrier function by using the intestinal permeability of FD4, a high molecular weight and hydrophilic model compound, as an index. Simultaneous administration of the DGAT inhibitors, but neither of them alone, significantly increased plasma FD4 concentration 5 hr after administration of FD4 compared with the vehicle (Figure 4).

## 4 DISCUSSION

**Figure 5:**
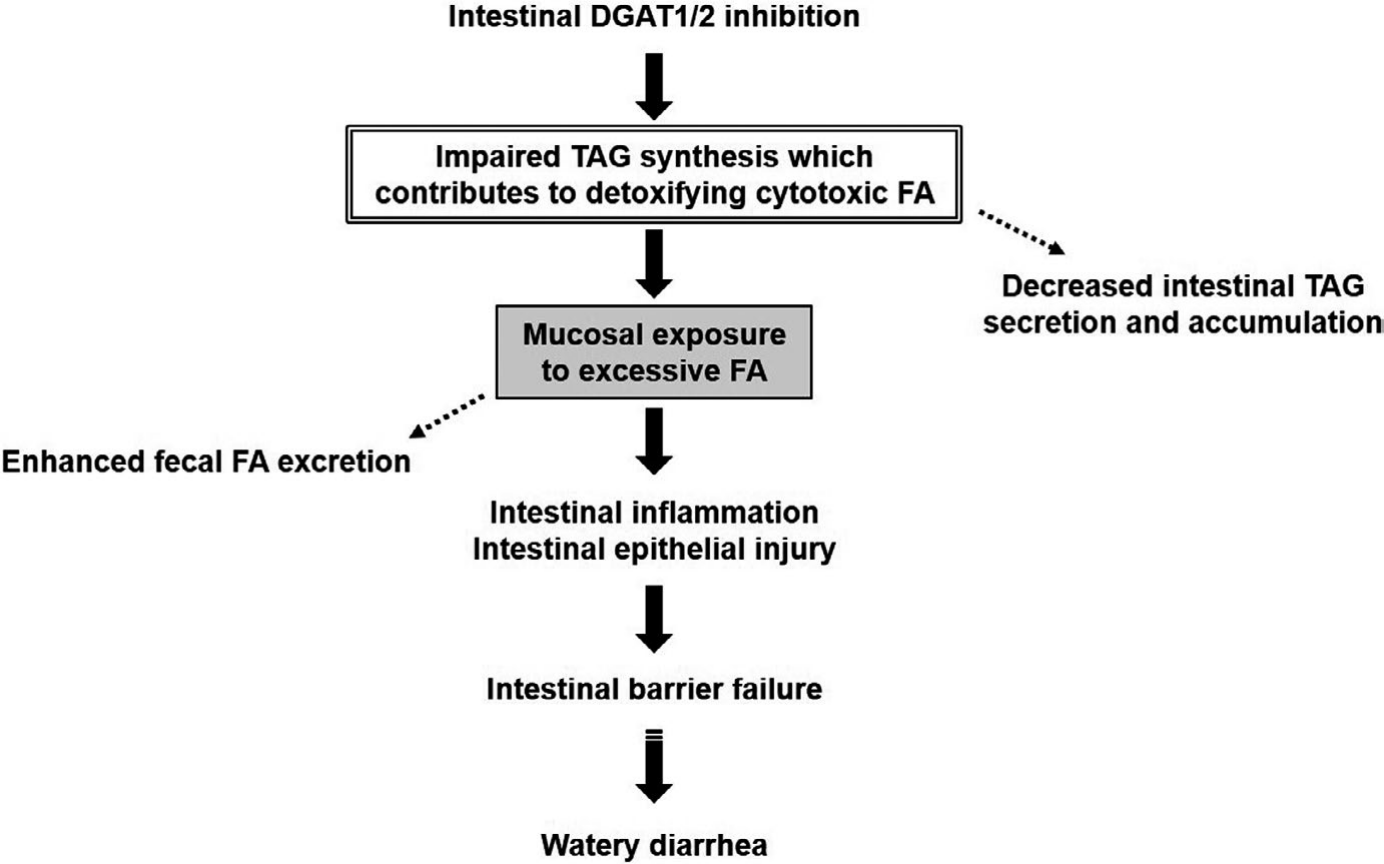
Proposed mechanisms by which DGAT1/2 inhibition leads to diarrhea in mice. In the intestine, DGAT1/2 inhibition impairs TAG synthesis which contributes to detoxification of cytotoxic FA and causes mucosal exposure to excess FA. As a result, the dysregulation of cytotoxic FA metabolism induces intestinal inflammation and intestinal injury, leading to intestinal barrier failure and subsequent diarrhea in DGAT1/2 inhibitor‐treated mice

The present study examined whether and how DGAT1 and DGAT2 inhibition, alone or in combination, leads to watery diarrhea in mice. Simultaneous inhibition of DGAT1 and DGAT2, but not DGAT1 or DGAT2 inhibition alone, elicited watery diarrhea only under HFD feeding in mice. We found that the DGAT1/2 inhibitor‐treated mice exhibited crypt hyperplasia and shortening of the villi in the duodenum and jejunum under HFD feeding. The intestinal pathological changes were accompanied by altered inflammatory responses, such as increased intestinal PGE_2_ and fecal calprotectin, indicating that the changes represent intestinal injury. The DGAT1/2 inhibitor‐treated mice also exhibited intestinal epithelial barrier failure, which was indicated by increased intestinal permeability of FD4, and luminal fluid accumulation. It has been reported that intestinal injury and inflammation contribute to impairment of the intestinal barrier function (Schulzke et al., 2009; Williams et al., 2013) and that intestinal barrier failure leads to impaired water transport and watery diarrhea by fluid exudation into the intestinal lumen (Musch et al., 2002; Schulzke et al., 2009). Therefore, it is likely that DGAT1/2 inhibition induced intestinal inflammation and injury and resulted in the intestinal barrier failure and subsequent watery diarrhea (Figure 5).

Decreased plasma and duodenal TAG and increased fecal FA following DGAT1/2 inhibition raise the possibility that DGAT1/2 inhibition caused a decrease in utilization of FA in enterocytes, resulting in intestinal mucosal exposure to excessive FA. An excessive amount of FA exerts cytotoxic effects on cells (Listenberger et al., 2003; Listenberger, Ory, & Schaffer, 2001) and high dietary intake of FA reportedly increases the risk of inflammatory bowel disease, characterized by chronic inflammation of the intestine (IBD in EPIC Study Investigators, 2009; Shores, Binion, Freeman, & Baker, 2011). Considering these findings, the intestinal mucosal exposure to excessive FA is highly likely to be a trigger for diarrhea induced by DGAT1/2 inhibition (Figure 5). It has been suggested that DGAT‐mediated TAG synthesis contributes to detoxification of cytotoxic FA in cells (Listenberger et al., 2003). In the duodenum and jejunum, the expressions of molecules involved in dietary FA uptake from the intestinal lumen are higher than in the ileum (Chen, Yang, Braunstein, Georgeson, & Harmon, 2001; Poirier, Degrace, Niot, Bernard, & Besnard, 1996). In the DGAT1/2 inhibitor‐treated mice, impaired TAG synthesis may result in insufficient detoxification of cytotoxic FA taken up by enterocytes because intestinal injury is observed only in the upper part of the small intestine (Figure 5).

The present study demonstrated that DGAT1 inhibitor‐treated mice appeared healthy in agreement with previous studies (Smith et al., 2000). Moreover DGAT1 inhibition alone inhibited plasma radiolabeled TAG after oral gavage of radiolabeled TAG, which was further decreased by DGAT1/2 inhibition. While duodenal radiolabeled TAG was not affected by either DGAT1 or DGAT2 inhibitor alone, it was significantly inhibited by DGAT1/2 inhibition. These results suggest that, in mouse small intestine, DGAT1 is the predominant DGAT in dietary TAG absorption and that DGAT2 plays a compensatory role when DGAT1 activity is inhibited. These findings further suggest that almost complete defects in TAG synthesis are associated with intestinal dysfunction such as barrier failure and diarrhea. We observed increased intracytoplasmic vacuoles in the jejunum of DGAT1 inhibitor‐treated mice under HFD feeding. The vacuoles are seemingly due to TAG accumulated in enterocytes because the histological images are similar to those of lipid droplets in the small intestine of DGAT1^‐/‐^ mice in previous reports (Buhman et al., 2002). Therefore, the morphological changes in DGAT1 inhibitor‐treated mice are not assumed to be involved in the pathogenesis of watery diarrhea.

In humans, treatment with a DGAT1 inhibitor alone leads to diarrhea with increased fecal calprotectin and FA (Denison et al., 2014), and a patient with loss‐of‐function mutation in DGAT1 exhibits severe diarrhea with shortening of the villi (Haas et al., 2012). These phenotypes may be strikingly similar to those of DGAT1/2 inhibitor‐treated mice. This is probably because only DGAT1 is highly expressed in the small intestine of humans unlike in mice (Cases et al., 1998, 2001; Haas et al., 2012). The diarrhea caused by genetic or pharmacological inhibition of DGAT1 in humans also could be attributed to the dysregulation of cytotoxic FA metabolism in the intestinal enterocytes.

In summary, our results demonstrate that a decrease in DGAT1 and DGAT2 activities leads to severe watery diarrhea in mice. We also suggest that dysregulation of cytotoxic FA metabolism is the key mechanism leading to diarrhea accompanied by intestinal barrier failure. Although further studies are needed to fully understand the exact mechanisms, such as changes in mucus layer, antimicrobial peptides and tight junction proteins, the present findings emphasize the vital importance of intestinal DGAT‐mediated TAG synthesis during the dietary TAG absorption process.

## CONFLICT OF INTERESTS

The authors declare that they have no competing interests.

## AUTHOR CONTRIBUTIONS

Kosuke Takemoto contributed to study concept and design, acquisition of data, analysis and interpretation of data, statistical analysis, drafting of the manuscript, critical revision of the manuscript for important intellectual content. Yumiko Fukasaka contributed to study concept and design, acquisition of data, analysis and interpretation of data. Ryo Yoshimoto contributed to drafting of the manuscript, critical revision of the manuscript for important intellectual content. Hirohide Nambu and Hideo Yukioka contributed to drafting of the manuscript.

## Supporting information



Fig S1

Fig S2

Fig S3

Fig S4

Supplementary Material
