# Supplementary figures and images for: Diacylglycerol acyltransferase 1/2 inhibition induces dysregulation of fatty acid metabolism and leads to intestinal barrier failure and diarrhea in mice
Source: Physiol Rep. 2020 Aug 12;8(15):e14542. doi: 10.14814/phy2.14542 (PMC7422801; doi:10.14814/phy2.14542)

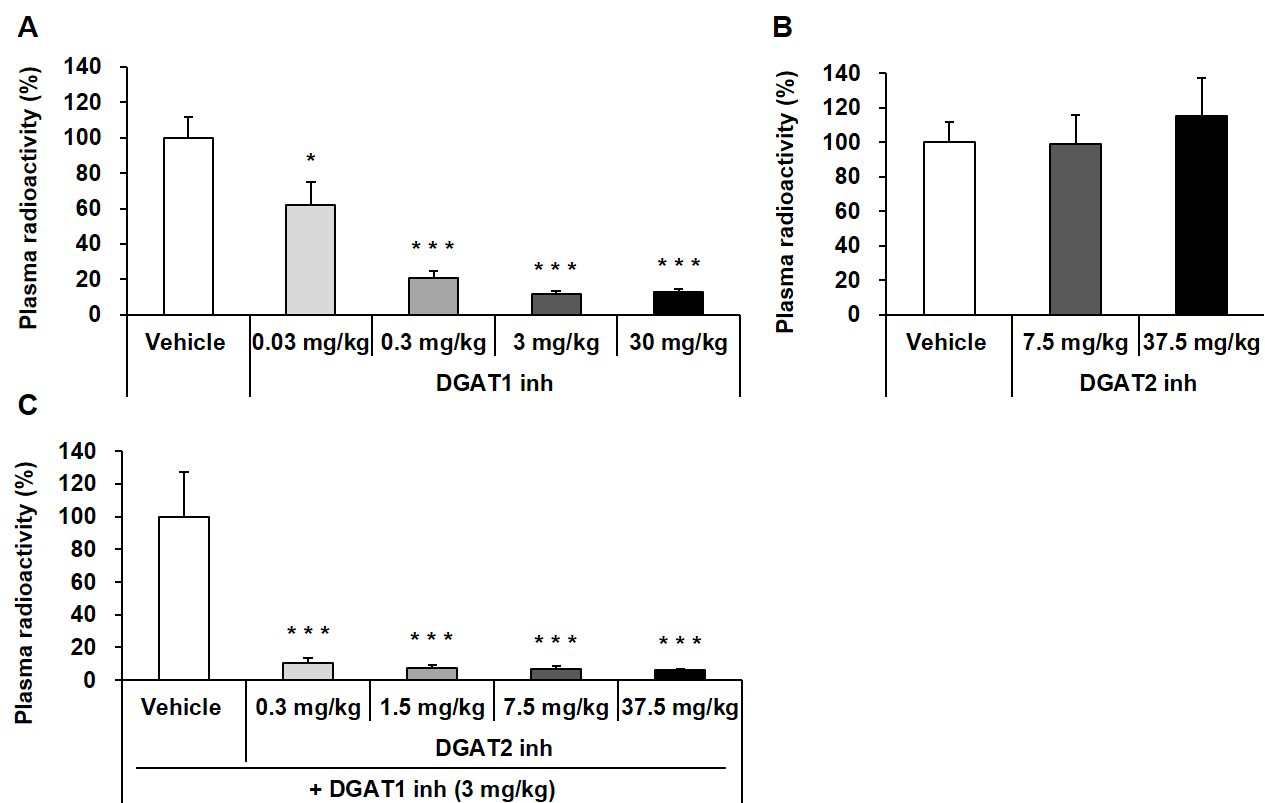

Supplement: Supplementary file 1 — Fig S1 [file PHY2-8-e14542-s001.tiff]

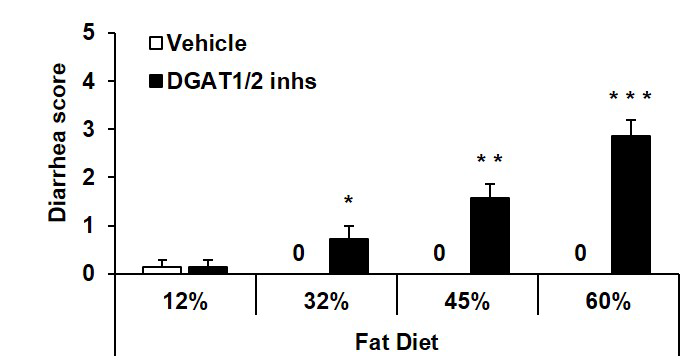

Supplement: Supplementary file 2 — Fig S2 [file PHY2-8-e14542-s002.tif]

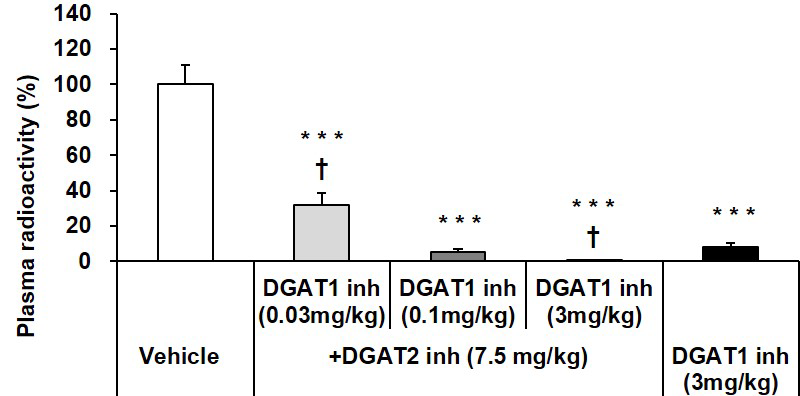

Supplement: Supplementary file 3 — Fig S3 [file PHY2-8-e14542-s003.tif]

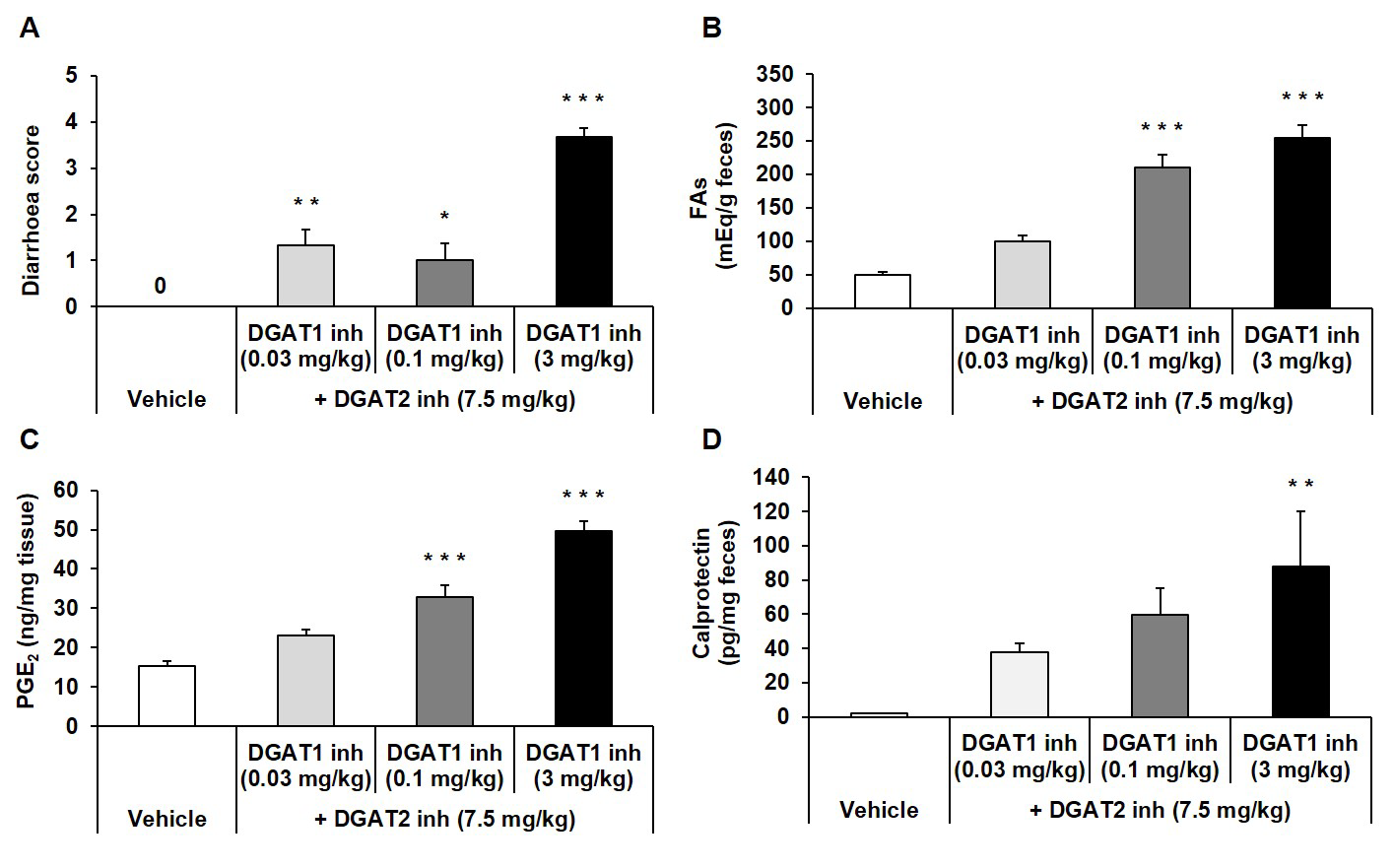

Supplement: Supplementary file 4 — Fig S4 [file PHY2-8-e14542-s004.tif]
