# Supplementary material for: Diacylglycerol acyltransferase 1/2 inhibition induces dysregulation of fatty acid metabolism and leads to intestinal barrier failure and diarrhea in mice
Source: Physiol Rep. 2020 Aug 12;8(15):e14542. doi: 10.14814/phy2.14542 (PMC7422801; doi:10.14814/phy2.14542)
Supplement: Supplementary file 5 — Supplementary Material [file PHY2-8-e14542-s005.docx]

**Diacylglycerol acyltransferase 1/2 inhibition induces dysregulation of fatty acid metabolism and leads to intestinal barrier failure and diarrhea in mice**

Kosuke Takemoto^1, 2,*^, Yumiko Fukasaka^1^, Ryo Yoshimoto^1^, Hirohide Nambu^1^, Hideo Yukioka^1^

^1^ Drug Discovery & Disease Research Laboratory, Shionogi & Co., Ltd., Osaka, Japan

^2^ Laboratory of Veterinary Pathology, Joint Faculty of Veterinary Medicine, Yamaguchi University, Yamaguchi, Japan

^*^To whom correspondence should be addressed

Address: Shionogi Pharmaceutical Research Center, 3-1-1, Futaba-cho, Toyonaka, Osaka 561-0825, Japan

Telephone: +81-6-6331-7632, Fax: +81-6-6332-6385

E-mail: [kosuke.takemoto@shionogi.co.jp](mailto:kosuke.takemoto@shionogi.co.jp)

**Running head**: DGAT1/2 inhibition leads to diarrhea in mice**Supplemental experimental procedures**

**Analysis of inhibitory action on intestinal TAG secretion**

Six-week-old male C57BL/6 mice were maintained on chow diet (CE-2; CLEA Japan). After at least 4 weeks of CE-2 feeding, mice were made to fast for 24 h prior to administration of vehicle or the DGAT inhibitors. At 15 minutes after the administration, 500 mg/kg of Pluronic F-127 was intraperitoneally injected to inhibit clearance of plasma TAG. At 15 minutes after the injection, 50 μCi/kg of [carboxyl-^14^C] triolein in 20% lipid emulsion was given by oral gavage. Blood samples were collected under anesthesia 60 min after the oral gavage, and plasma radioactivity was quantified by measuring ionizing radiation with a MicroBeta TRILUX counter.

**Analysis of dietary lipid-dependent effects**

Six-week-old male C57BL/6 mice were maintained on CE-2 (12% kcal fat) or HFDs containing 32% kcal fat (D12266B, Research Diets), 45% kcal fat (D12451, Research Diets) and 60% kcal fat for 4 weeks. Vehicle or the DGAT inhibitors were orally administered twice daily to mice for 2 days. Diarrhea scores were determined in the same manner as described in the main text.

**Supplemental figure legends**

**Figure S1.** Plasma radioactivity after oral gavage of [^14^C] TAG in DGAT1 inhibitor-treated groups (A), DGAT2 inhibitor-treated groups (B) and simultaneous treatment groups (C). DGAT1 and DGAT2 inhibitors, alone or in combination, were orally administered to fasted mice prior to oral gavage of lipid emulsion containing 50 μCi/kg of [^14^C] TAG. Plasma radioactivity was measured at 60 min after the oral gavage. The results are expressed as the percentage of the vehicle-treated group and the mean ± SEM ((A-B) n = 4, (C) n = 5). ^*^*P*<0.05, ^***^*P*<0.001 vs. Vehicle group (Dunnett's multiple-comparison test).

**Figure S2.** Dietary lipid-dependent effects of DGAT inhibitors on diarrhea scores in mice fed CE-2 (12% kcal fat) or HFDs (32, 45, or 60% kcal fat). DGAT1 inhibitor (3 mg/kg) and DGAT2 inhibitor (7.5 mg/kg) in combination were orally administered to CE-2 or HFDs fed mice for 2 days. All feces were collected during the period, and diarrhea scores were evaluated by measuring the softness and appearance of each fecal sample. The results are expressed as the mean ± SEM (n = 7). ^*^*P*<0.05, ^**^*P*<0.01, ^***^*P*<0.001 vs. Vehicle group (Welch’s t-test)

**Figure S3.** Dose-dependent effects of DGAT inhibitors on plasma radioactivity after oral gavage of [^14^C] TAG to mice. A DGAT1 inhibitor (0.03, 0.1, 3 mg/kg) and DGAT2 inhibitor (7.5 mg/kg) in combination or a DGAT1 inhibitor (3 mg/kg) alone were orally administered to fasted mice prior to oral gavage of lipid emulsion containing 50 μCi/kg of [^14^C] TAG. Plasma radioactivity was measured at 60 min after the oral gavage. The results are expressed as the percentage of the vehicle-treated group and the mean ± SEM (n = 5). ^***^*P*<0.001 vs. Vehicle group (Dunnett's multiple-comparison test)., ^†^*P*<0.05 vs. DGAT1 inhibitor-treated group (Welch’s t-test).

**Figure S4.** Dose-dependent effects of DGAT inhibitors on diarrhea score (A), fecal FA levels (B), jejunal PGE_2_ levels (C) and fecal calprotectin levels (D) in 60% HFD fed mice. A DGAT1 inhibitor (0.03, 0.1, 3 mg/kg) and DGAT2 inhibitor (7.5 mg/kg) in combination were orally administered to 60% HFD fed mice for 2 or 3 days. All feces were collected during the period, and diarrhea scores were evaluated by measuring the softness and appearance of each fecal sample. Fecal FA levels were determined by enzymatic methods, and levels of intestinal PGE_2_ and fecal calprotectin were determined with ELISA kits. The results are expressed as the mean ± SEM (n = 6). ^*^*P*<0.05, ^**^*P*<0.01, ^***^*P*<0.001 vs. Vehicle group (Dunnett's multiple-comparison test).
